# Retraction: Honeycomb-like Co–B amorphous alloy catalysts assembled by a solution plasma process show enhanced catalytic hydrolysis activity for hydrogen generation

**DOI:** 10.1039/d0ra90028a

**Published:** 2020-03-27

**Authors:** Dong Ge Tong

**Affiliations:** Mineral Resources Chemistry Key Laboratory of Sichuan Higher Education Institutions, College of Materials and Chemistry & Chemical Engineering, Chengdu University of Technology Chengdu 610059 China tongdongge@163.com +86-28-8407-9074; Institute of Green Catalysis and Synthesis, College of Materials and Chemistry & Chemical Engineering, Chengdu University of Technology Chengdu 610059 China

## Abstract

Retraction of ‘Honeycomb-like Co–B amorphous alloy catalysts assembled by a solution plasma process show enhanced catalytic hydrolysis activity for hydrogen generation’ by Dong Ge Tong *et al.*, *RSC Adv.*, 2012, **2**, 2369–2376.

We, the authors, hereby wholly retract this article due to inaccuracies in the scale bars of Fig. 1b, 4h and 5h and the wrong images being presented in Fig. 5d and e.

We, the authors, repeated the experiments and believe that the scientific content and conclusions of the related studies presented in the published paper can be reproduced. However, due to the large number of images, it is not possible to replace the published images with the new figures. Therefore, to protect the rigor of the scientific record, we decided to retract this article on our own initiative.

Signed: Dong Ge Tong (on behalf of the authors).

Date: 6^th^ March 2020.

Retraction endorsed by Laura Fisher, Executive Editor, *RSC Advances*.

## Supplementary Material

